# Effects of Micronutrient Supplementation on Glucose and Hepatic Lipid Metabolism in a Rat Model of Diet Induced Obesity

**DOI:** 10.3390/cells10071751

**Published:** 2021-07-11

**Authors:** Saroj Khatiwada, Virginie Lecomte, Michael F. Fenech, Margaret J. Morris, Christopher A. Maloney

**Affiliations:** 1 School of Medical Sciences, UNSW Sydney, Sydney, NSW 2052, Australia; s.khatiwada@unsw.edu.au (S.K.); virginie.lecomtemaloney@uts.edu.au (V.L.); m.morris@unsw.edu.au (M.J.M.); 2 School of Pharmacy and Medical Sciences, University of South Australia, 108 North Terrace, Adelaide, SA 5001, Australia; michael.fenech@unisa.edu.au

**Keywords:** antioxidants, glucose metabolism, liver, micronutrients, obesity, oxidative stress

## Abstract

Obesity increases the risk of metabolic disorders, partly through increased oxidative stress. Here, we examined the effects of a dietary micronutrient supplement (consisting of folate, vitamin B6, choline, betaine, and zinc) with antioxidant and methyl donor activities. Male Sprague Dawley rats (3 weeks old, 17/group) were weaned onto control (C) or high-fat diet (HFD) or same diets with added micronutrient supplement (CS; HS). At 14.5 weeks of age, body composition was measured by magnetic resonance imaging. At 21 weeks of age, respiratory quotient and energy expenditure was measured using Comprehensive Lab Animal Monitoring System. At 22 weeks of age, an oral glucose tolerance test (OGTT) was performed, and using fasting glucose and insulin values, Homeostasis Model Assessment of Insulin Resistance (HOMA-IR) was calculated as a surrogate measure of insulin resistance. At 30.5 weeks of age, blood and liver tissues were harvested. Liver antioxidant capacity, lipids and expression of genes involved in lipid metabolism (*Cd36*, *Fabp1*, *Acaca*, *Fasn*, *Cpt1a*, *Srebf1*) were measured. HFD increased adiposity (*p* < 0.001) and body weight (*p* < 0.001), both of which did not occur in the HS group. The animals fed HFD developed impaired fasting glucose, impaired glucose tolerance, and fasting hyperinsulinemia compared to control fed animals. Interestingly, HS animals demonstrated an improvement in fasting glucose and fasting insulin. Based on insulin release during OGTT and HOMA-IR, the supplement appeared to reduce the insulin resistance developed by HFD feeding. Supplementation increased hepatic glutathione content (*p* < 0.05) and reduced hepatic triglyceride accumulation (*p* < 0.001) regardless of diet; this was accompanied by altered gene expression (particularly of *CPT-1*). Our findings show that dietary micronutrient supplementation can reduce weight gain and adiposity, improve glucose metabolism, and improve hepatic antioxidant capacity and lipid metabolism in response to HFD intake.

## 1. Introduction

Obesity has emerged as a significant health issue across the world, including Australia, increasing the risk for metabolic diseases, mental disorders, reproductive disorders, and cancers [1]. Increased oxidative stress has been implicated as a common factor for most of the obesity-related metabolic complications such as prediabetes, insulin resistance, and type 2 diabetes mellitus (T2DM) [2,3]. Therefore, reducing oxidative stress may help to reduce obesity-related metabolic disorders.

A number of micronutrients have antioxidant properties and are the key molecules that can prevent the generation of oxidative stress. Micronutrients are generally consumed in small amounts in the diet, yet they are essential and have critical roles in metabolic reactions [4]. In this current study, we investigated the benefits of supplementing semisynthetic control or high fat diets (HFD) with a unique formulation comprising five micronutrients: folate, vitamin B6, choline, betaine, and zinc (ZnSO_4_). These components were chosen as they participate in one-carbon metabolism, providing substrates for glutathione synthesis, either as a substrate or a cofactor [5], and themselves act as antioxidants [6]. Moreover, additional vitamin B6 in the formulation can increase glutathione synthesis, through trans-sulphuration pathway enhancing antioxidant status [7]. Although these components have been extensively investigated for their role in epigenetic processes, as methyl donor precursors, here we wished to investigate their anti-oxidative effects [8]. Furthermore, oxidation may cause gene silencing by causing damage to promoter regions of genes [9]. Therefore, the supplement antioxidant effects may also prevent this less known epigenetic drift aspect.

Several previous animal studies have supplemented one of the micronutrients applied here and investigated the effects on glucose metabolism and liver. There is some evidence for improvement in glucose metabolism [10], insulin resistance [11], and hepatic oxidative stress [12] by folate supplementation. Vitamin B6 [13], betaine [14], and choline [15] supplementation can reduce oxidative stress, hepatic pathological changes, and weight gain respectively. Zinc supplementation has been shown to reduce insulin resistance in people with obesity [16].

There is little evidence about the benefits of supplementing multiple micronutrients that participate in one-carbon metabolism. Only three animal studies incorporating three (folate 5.5 mg/kg, vitamin B12 0.5 mg/kg, betaine 5 g/kg and choline 5.37 g/kg) [17,18] or four (folate 15 mg/kg, vitamin B12 1.5 mg/kg, betaine 15 g/kg, choline 15 g/kg, methionine 7.5 g/kg and ZnSO_4_ 150 mg/kg) [19] components of our micronutrient formulation have been published, focusing mainly on hepatic lipid metabolism. To our knowledge the impact of increased vitamin B6, combined with these other micronutrients has not been investigated previously.

In this study, we hypothesized that the protection of male rats from HFD induced oxidative stress, by consuming a dietary micronutrient supplement would improve glucose metabolism, hepatic oxidative stress, and hepatic lipid metabolism. Interestingly, significant benefits of micronutrient supplement on metabolic health, and hepatic antioxidant capacity, and lipid metabolism were seen.

## 2. Materials and Methods

### 2.1. Animals

Male Sprague Dawley (SD) pups generated in the Biological Resources Centre, UNSW SYDNEY (Founders sourced from ARC, Perth, WA) were maintained at 21 ± 2 °C; 12:12 h light/dark condition. The use of animals for this research and all experimental procedures were approved by the Animal Ethics Committee of the University of New South Wales (Ethics ID: 14/44B and 17/27A).

**Figure 1:**
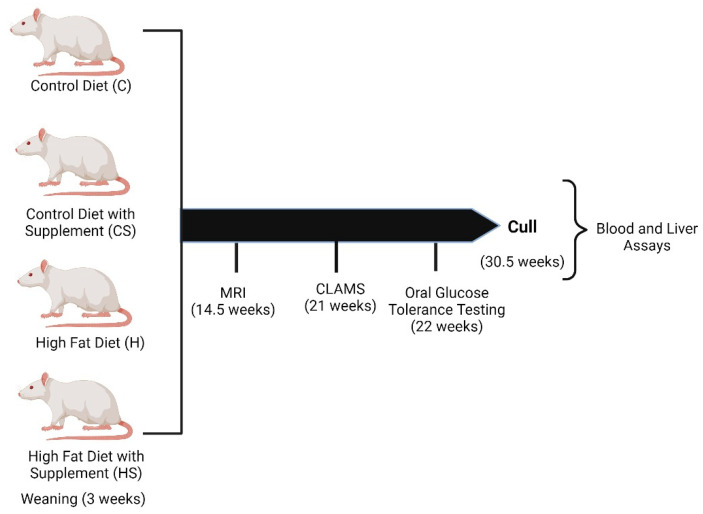
Experimental design and timeline.

At 3 weeks of age, animals were housed 2–3 rats per cage and fed one of the four diets (17 animals in each diet group) until cull. The diets used were control diet (denoted by C), control diet containing micronutrient supplement (denoted by CS), HFD (denoted by H) and HFD containing micronutrient supplement (denoted by HS). In order to maintain a body weight difference between control fed and HFD fed animals while maintaining health, we limited the energy intake of the C and CS groups from the age of 7 weeks (from 4 weeks of diet) to 407 kJ (32 g/rat/day of diet). This amount of energy intake corresponds to daily recommended intake for rats, and our long experience with adult SD male rats suggests this energy intake is sufficient for normal growth [20]. EchoMRI was performed at 14.5 weeks of age, and blood samples (data not shown) were collected at 15 weeks of age. The animals were mated at 19 weeks of age with control fed females to generate F1 offspring (not discussed here). The animal’s respiratory quotient (RQ) and energy expenditure was measured at 21 weeks using Comprehensive Lab Animal Monitoring System (CLAMS). Animals underwent oral glucose tolerance testing (OGTT) at 22 weeks of age before being culled at 30.5 weeks of age. Blood, and hepatic tissues were collected and frozen at cull. The experimental design is shown in Figure 1.

The animals were weaned at 3 weeks of age to one of four diets (C, CS, H or HS). The animals underwent magnetic resonance imaging (MRI), respiratory quotient and energy expenditure measurement using Comprehensive Lab Animal Monitoring System (CLAMS) and oral glucose tolerance test at 14.5, 21 and 22 weeks respectively. Endpoint blood and liver tissues were collected at the age of 30.5 weeks and biochemical and molecular assays were performed.

### 2.2. Diets

The diets were prepared in-house using commercial ingredients, and the micronutrient supplements were added during preparation. The composition of both control diets/HFDs were similar except for sucrose, canola oil, lard, corn starch and supplemented micronutrients content as shown in Table 1. Control diet and HFD were prepared based on American Institute of Nutrition 93 G/M (AIN 93 G/M) formulation [21], and SF03-020 (Specialty Feeds, Western Australia, containing 23% total fat and 20 MJ/kg digestible energy) respectively. The energy content of our control and control supplemented diet was 12,720 kJ/kg diet, and that of HFD and HFD supplemented diet was 20,439 kJ/kg diet.

The micronutrients formulation for the supplementation was designed based on the Wolff 3SMZ diet used in mice except the increase in vitamin B12 was replaced by additional B6 and methionine was given at normal levels [22]. Their 3SMZ diet contained five times the amount of choline, betaine, folic acid and zinc present in a typical AIN93M diet. The dose of vitamin B6 in our supplement was determined based upon a previous study which used a diet containing vitamin B6 at 30 mg/kg of diet [23].

### 2.3. EchoMRI

At the age of 14.5 weeks (11.5 weeks of diet), MRI scans were taken using EchoMRI™ (Houston, TX, USA) whole body composition analyser to measure fat and lean mass.

### 2.4. Respiratory Quotient Measurement

At 21 weeks of age, animals were placed in a Comprehensive Lab Animal Monitoring System (CLAMS, Columbus Instruments, Columbus, OH, USA) to measure basal energy expenditure and RQ values. Animals were monitored during the light phase with ad libitum access to water but not food to determine basal metabolic rate. After this period, animals had ad libitum access to their corresponding diets. Both RQ, and index of basal metabolic rate (kJ/h) were determined at 30 min intervals and the values were averaged. The CLAMS also provided information on animal’s activity, food and water consumption.

### 2.5. Glucose Tolerance Testing

Glucose tolerance was assessed by OGTT; briefly, animals were fasted overnight and loaded with 50% glucose solution (dose 2 g/kg body weight) by oral gavage. Fasting blood glucose and the blood glucose after gavage were measured at 15, 30, 45, 60, 90 and 120 min using Accu-Chek glucometer (Roche Diagnostics, Castle Hill, NSW, Australia). Insulin levels at fasting and time points 15, 30, 60 and 120 min of OGTT were determined via ELISA (Crystal Chem, Elk Grove Village, IL, USA). Fasting glucose and insulin values were used to calculate Homeostasis Model Assessment of Insulin Resistance (HOMA-IR) using the equation [24]:

(1) $$HOMA-IR=\frac{\left[fasting\;insulin\;\left(ng/mL\right)\times fasting\;glucose\;\left(mM\right)\right]}{22.5\times 0.0403}$$

This value was used as a proxy measure of insulin resistance in rats [25]. The area under the curve (AUC) for glucose and insulin concentration during OGTT was calculated (using time points 0–120 min).

### 2.6. Plasma Concentration of Insulin, Leptin, Lipids and TBARS

Plasma concentrations of insulin and leptin were measured using commercial ELISA kits (Ultra-Sensitive Rat Insulin and Leptin ELISA kit, Crystal Chem, Elk Grove Village, IL, USA). The sensitivity, linearity range, intra- and inter-assay precision CV of insulin kit was 0.05 ng/mL, 0.1–12.8 ng/mL, ≤10% and ≤10% respectively. For leptin kit, the sensitivity, linearity range, intra- and inter-assay precision CV was 200 pg/mL, 0.2–12.8 ng/mL, ≤10% and ≤10% respectively. Triglyceride (TG) and total cholesterol concentrations were measured in plasma using Roche triglyceride reagent and cholesterol reagent from Thermo Scientific (Waltham, MA, USA) respectively. The sensitivity, linearity range, intra- and inter-assay precision CV of triglyceride kit was 0.002 ∆A per mg/dL (1 cm light path, 500 nm), 0–885 mg/dL, 2.07% and 4.5% respectively. For total cholesterol, the sensitivity, linearity range, intra- and inter-assay precision CV was 1.6 ∆mAbs per mg/dL (1 cm light path, 500 nm), 0–774 mg/dL, 2.8 and 2.8% respectively. Plasma oxidative stress was estimated by measuring a marker of lipid peroxidation, thiobarbituric acid reactive substance (TBARS) using colorimetric method (OxiSelect™ TBARS Assay Kit from Cell Biolab Inc., San Diego, CA, USA).

### 2.7. Hepatic Folate, TBARS and Glutathione Content

For hepatic folate and TBARS measurement, liver was homogenized in phosphate buffered saline (PBS). Folate concentration in the supernatant was estimated by ELISA method (Monobind Inc., Lake Forest, CA, USA) and TBARS by method described for plasma above. For folate kit, the sensitivity, linearity range, intra- and inter-assay precision CV was 0.52 ng/mL, 0–25 ng/mL, <10%, and <10% respectively. Hepatic antioxidant status was estimated by measuring total glutathione concentration in liver extract. For this, liver powder was homogenized using 5% sulphosalicyclic acid (SSA). Total glutathione concentration was estimated in the supernatant by kinetic assay using Ellman’s reagent (kits from Sigma-Aldrich, St. Louis, MO, USA). All the tissue measures were normalized to the weight of tissue assayed.

### 2.8. Hepatic Lipid Extraction and Lipid Assays

Hepatic lipids were extracted from ground liver powder based on Folch method [26]. The extract was dissolved in absolute ethanol, and triglyceride and total cholesterol concentrations were measured as described above and normalized to the weight of tissue assayed.

### 2.9. Hepatic RNA Extraction and Measurement of Gene Expression

Total RNA was extracted from liver, and cDNA was synthesized as described previously [27]. RT-qPCR was performed on the QuantStudio 12K Flex (Life Technologies, Carlsbad, CA, USA) as described previously [27], and in compliance with the MIQE guidelines [28]. Initially, reference genes (Table 2) were screened based on stability values (eight samples per group were tested) using R software (RStudio Team, Boston, MA, USA) and the best combination of genes was selected [29]. The most stable genes were found to be *B2M* and *HPRT-1*. Expression of genes involved in fatty acid uptake (*Cd36*), intracellular binding (*Fabp1*), synthesis (*Acaca* and *Fasn*) and breakdown (*Cpt1a*) of fatty acids were measured using RT-PCR. Furthermore, expression of Srebf1, which regulates the activity of lipogenic genes, and *Hnf4a*, which plays a role in hepatic differentiation were measured. Genes selected for RT-qPCR and the corresponding hydrolysis probe references (Life Technologies, CA, USA) are listed in Table 2. Relative gene expression was calculated using the 2^−ΔΔCT^ method, normalised against two reference genes and an external calibrator [30].

### 2.10. Statistical Analysis

Initially, all the data were checked for normality using the Shapiro-Wilk normality test. Normally distributed variables were presented as mean ± SEM. Statistical analyses were performed using the IBM SPSS v23.0 software (IBM Corp, Armonk, NY, USA). Data were analysed by two-way ANOVA followed by post hoc LSD for single measurements. Where appropriate repeated measures two-way ANOVA was conducted (energy intake, body weight, OGTT) followed by LSD post hoc. The main effects followed by the post hoc LSD were considered if there were no interaction effects. In the case of interaction of HFD and supplement, simple main effect analysis was performed, and the resulting post hoc from interaction effect was considered.

Overall HFD effect and overall supplement effect are denoted by ‘*’, and ‘#’. The post hoc HFD effect and supplement effect are represented by alphabet ‘a’ (HFD effect, ‘H’ versus ‘C’), ‘b’ (HFD effect, ‘HS’ versus ‘CS’), ‘c’ (supplement effect, ‘CS’ versus ‘C’), and ‘d’ (supplement effect, ‘HS’ versus ‘H’). Differences were considered significant at *p* < 0.05. The reported error bars in text and figures are SEM.

## 3. Results

All the animals on different diets grew normally and no visible adverse effects were associated with HFD or micronutrient supplementation.

### 3.1. Body Weight, Adiposity and Respiratory Quotient

**Figure 2:**
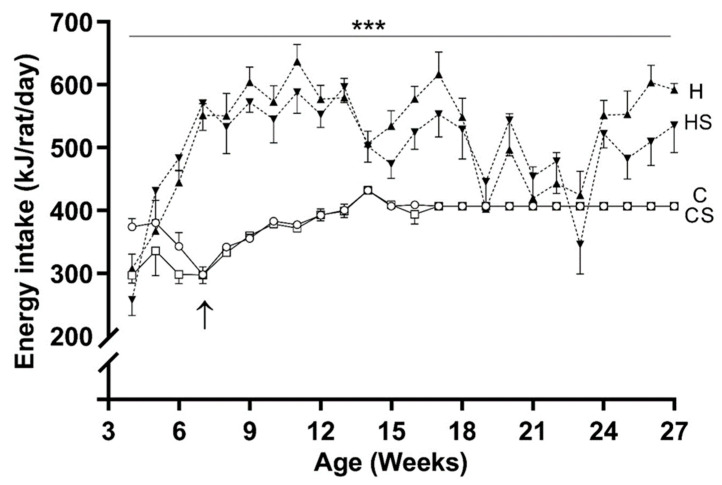
Energy intake from weaning until 27 weeks. Data are shown as mean ± SEM (*n* = 17 per group) in rats consuming C (solid lines and open circle), CS (solid lines and rectangle), H (dotted lines and upright triangle) and HS (dotted lines and inverted triangle) diets. The arrow (↑) indicates start of pair-feeding in C and CS groups at 7 weeks of age. Abbreviations: C, Control diet; CS, Control diet with supplement; H, HFD; and HS, HFD with supplement. Effects of HFD and supplement were assessed by repeated measures two-way ANOVA followed by LSD post hoc. Overall HFD effect *** *p* < 0.001.

**Figure 3:**
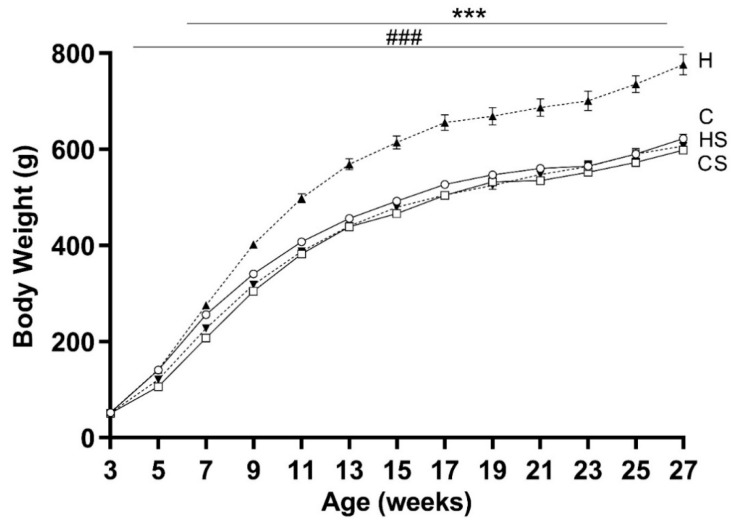
Body weight from weaning till 27 weeks. Data are shown as mean ± SEM (*n* = 17 per group) in rats consuming C (solid lines and open circle), CS (solid lines and rectangle), H (dotted lines and upright triangle) and HS (dotted lines and inverted triangle) diets. Abbreviations: C, Control diet; CS, Control diet with supplement; H, HFD; and HS, HFD with supplement. Effects of diet and supplement were assessed by repeated measures two-way ANOVA followed by LSD post hoc. There was an interaction effect (*p* < 0.001) of HFD and supplement for the whole period of animal growth. Overall HFD effect *** *p* < 0.001. Overall supplement effect ^###^ *p* < 0.001.

The weekly energy intake of H and HS groups were significantly higher than C and CS groups (*p* < 0.001), respectively. There were no differences in energy intake among H- and HS-fed animals (Figure 2). At the start of the dietary intervention (weaning), body weights across all four groups (C: 52.5 ± 1.4; CS: 50.8 ± 1.4; H: 52.8 ± 1.3 and HS: 51.3 ± 1.4 g) were similar. Overall, HFD promoted weight gain (*p* < 0.001, H versus C group), and supplement in HFD prevented weight gain (*p* < 0.001, HS versus H group) despite their similar energy intake (Figure 3). Supplementation in control diet fed animals (CS) did not affect their growth, with evolution of body weight from 3 to 27 weeks of age similar to the control group. At 14.5 weeks, the percent fat mass among the four diet groups were C: 13.1 ± 0.7, CS: 11.0 ± 0.5, H: 20.0 ± 1.0 and HS: 12.1 ± 0.8% respectively. Body fat percentage was higher in H group than C group (by 53%, *p* < 0.001) and HS group (by 65%, *p* < 0.001). At 21 weeks of age, the average RQ values among the groups were C: 0.845 ± 0.02, CS: 0.837 ± 0.02, H: 0.775 ± 0.02 and HS: 0.750 ± 0.01 respectively. An overall effect of HFD (*p* < 0.001) on RQ values was observed, with higher RQ in C group than H group (by 9%, *p* < 0.01) and in CS group than HS group (by 11.6%, *p* < 0.01). Micronutrient supplementation had no significant effects on RQ values. The basal metabolic rates were C: 11.6 ± 0.5, CS: 12.5 ± 0.5, H: 13.2 ± 0.5 and HS: 11.6 ± 0.3 kJ/h respectively. An interaction effect (*p* < 0.01) of HFD and supplement was seen on the basal metabolic rate. Basal metabolic rate was higher in the H group as compared to C group (*p* < 0.01), and HS group (*p* < 0.01).

### 3.2. Fasting Blood Glucose and Glucose Tolerance

**Figure 4:**
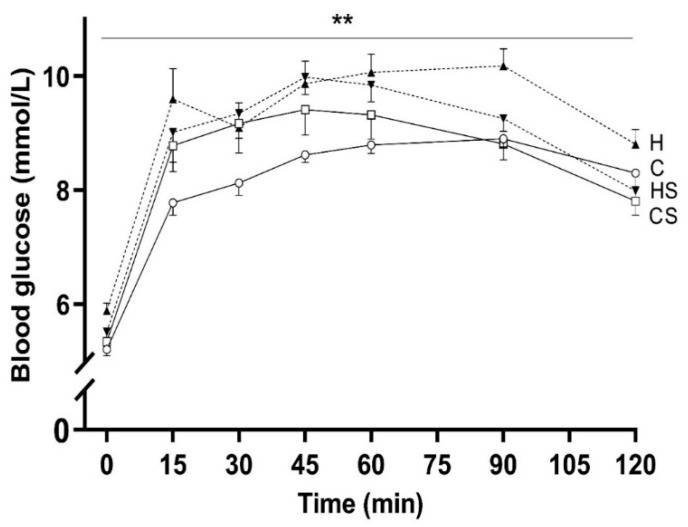
Blood glucose during OGTT. Data are shown as mean ± SEM (*n* = 15–16 per group) in rats consuming C (solid lines and open circle), CS (solid lines and rectangle), H (dotted lines and upright triangle) and HS (dotted lines and inverted triangle) diets. Abbreviations: C, Control diet; CS, Control diet with supplement; H, HFD; and HS, HFD with supplement. Effects of HFD and supplement were assessed by repeated measures two-way ANOVA followed by LSD post hoc. Overall HFD effect ** *p* < 0.01.

HFD fed animals had a mildly impaired fasting blood glucose (*p* < 0.001, H versus C group) which HS diet-fed animals did not develop (*p* = 0.042, HS versus H group; Table 3). Animals consuming HFD had higher blood glucose concentrations at several timepoints (15, 45, 60 and 90 min) during the OGTT as compared to C group (*p* = 0.002 by repeated measures analysis of blood glucose concentrations during OGTT). Supplement had no significant effect on blood glucose concentrations during OGTT (Figure 4). The AUC for blood glucose concentration across 120 min was higher in H group as compared to C group (*p* < 0.01).

### 3.3. Fasting Insulin and Insulin Sensitivity

**Figure 5:**
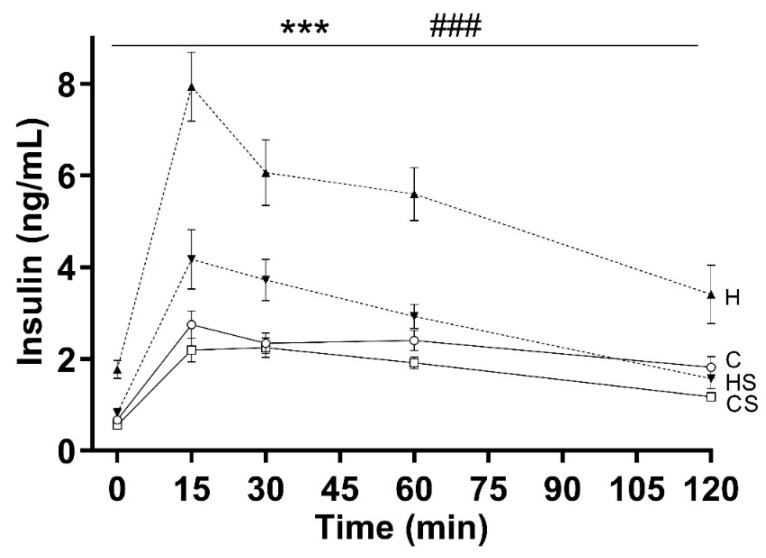
Insulin levels during OGTT. Data are shown as mean ± SEM (*n* = 15 per group) in rats consuming C (solid lines and open circle), CS (solid lines and rectangle), H (dotted lines and upright triangle) and HS (dotted lines and inverted triangle) diets. Abbreviations: C, Control diet; CS, Control diet with supplement; H, HFD; and HS, HFD with supplement. Effects of HFD and supplement were assessed by repeated measures two-way ANOVA followed by LSD post hoc. There was an interaction effect (*p* < 0.01) of HFD and supplement for the insulin release during OGTT. Overall HFD effect *** *p* < 0.001. Overall supplement effect ^###^ *p* < 0.001.

The H group had higher fasting insulin as compared to C group (*p* < 0.001, H versus C group), and HS group (*p* < 0.001, HS versus H group; Table 3). HOMA-IR values were higher in H group compared to C group (*p* < 0.001), and HS group (*p* < 0.001) as shown in Table 3. Both HFD (*p* < 0.001) and supplement (*p* < 0.001) showed effects on insulin release during OGTT, along with an interaction effect (*p* = 0.002) as shown in Figure 5. Furthermore, HFD fed animals released more insulin over several timepoints (15, 30, 60, 120 min) of OGTT than C group (*p* < 0.001 by repeated measures analysis), and HS group (*p* < 0.001 by repeated measures analysis; Figure 5). The AUC for insulin release was higher in H group as compared to C (*p* < 0.001) and HS (*p* < 0.001) group (Table 3).

### 3.4. Plasma Lipids, Leptin and TBARS

At endpoint (30.5 weeks of age), plasma triglyceride was higher in H as compared to C group (*p* = 0.007), and in HS compared to CS group (*p* = 0.003; Table 3). CS group had higher total cholesterol than C group (*p* < 0.001), and a trend (*p* = 0.053) of higher cholesterol was seen in H group as compared to the C group. There was no difference in the plasma TBARS level across the four groups (Table 3). The plasma leptin was higher in HFD group than C group (*p* < 0.001, H versus C group), and HS group (*p* < 0.001, H versus HS; Table 3).

### 3.5. Hepatic Antioxidant Capacity

In the liver, folate levels were higher in supplemented than non-supplemented animals (CS versus C; *p* = 0.002, and HS versus H; *p* = 0.032; Table 4). Total glutathione level was increased by the supplement (*p* = 0.010 overall effect, *p* = 0.057 for CS versus C, *p* = 0.071 for HS versus H). Hepatic TBARS levels were not significantly affected by HFD or supplement (Table 4).

### 3.6. Hepatic Lipids and Expression of Genes Involved in Lipid Metabolism

HFD animals had increased (*p* = 0.025), whereas supplemented animals had reduced (*p* < 0.001) hepatic triglyceride content (Table 4), compared to control group. Hepatic triglyceride content was higher in H group as compared to C group (higher by 44%, *p* = 0.009), and HS group (higher by 89%, *p* < 0.001), but lower in CS group as compared to C group (lower by 50%, *p* = 0.046). The HFD (*p* = 0.041) increased hepatic total cholesterol content; HS group had higher total cholesterol content than CS group (*p* = 0.011).

**Figure 6:**
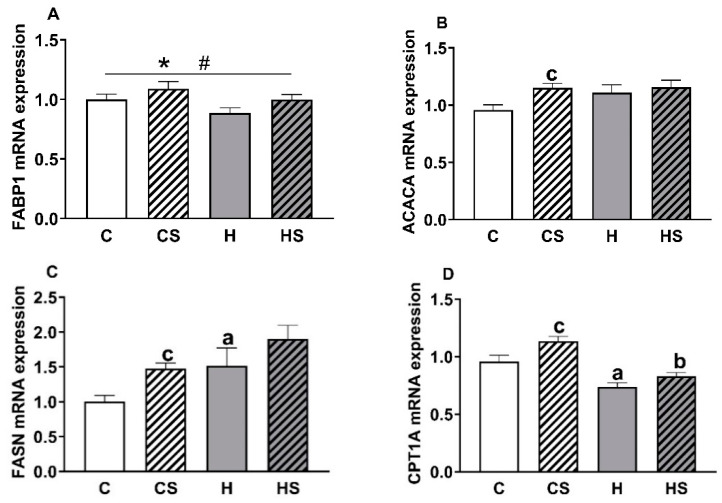
Expression of liver genes involved in lipid metabolism. Data are shown as mean ± SEM (*n* = 14–16 per group); panels A, B, C and D show mRNA expression of *FABP1, ACACA, FASN* and *CPT1A* genes respectively. Abbreviations: C, Control diet; CS, Control diet with supplement; H, HFD; and HS, HFD with supplement. Effects of HFD and supplement were assessed by two-way ANOVA followed by LSD post hoc. Post hoc HFD effect ‘a’ (H versus C) and ‘b’ (HS versus CS), and supplement effect ‘c’ (CS versus C). Overall HFD effect * *p* < 0.05, *p* < 0.01 or *p* < 0.001. Overall supplement effect # *p* < 0.05 or *p* < 0.01.

Overall, the expression of *Fabp1* gene was reduced in the HFD animals (H plus HS group versus C plus CS group, *p* = 0.043) and increased in the supplement group (CS plus HS group versus C plus H group, *p* = 0.047; Figure 6A). HFD feeding markedly increased the expression of *Fasn* gene (*p* = 0.007; Figure 6C) but not *Acaca* gene. The *Fasn* gene expression was significantly greater in H versus C rats (*p* = 0.035) with a trend (*p* = 0.079) towards higher expression in HS versus CS rats. Overall, feeding the supplement resulted in an increased expression of *Acaca* (*p* = 0.033; Figure 6B) and *Fasn* (*p* = 0.014); the CS group had higher expression of *Acaca* (*p* = 0.015), and *Fasn* (*p* = 0.048) gene than the C group. A significant effect of both HFD (*p* < 0.001) and supplement (*p* = 0.002) was seen for the expression of *Cpt1a*, which is involved in fatty acid catabolism (Figure 6D). The most marked effect was a reduction in *Cpt1a* expression by HFD in both H and HS groups relative to their controls (H versus C group; *p* = 0.001, and HS versus CS group; *p* < 0.001), and an increase in the CS compared to C group (*p* = 0.005). There were no differences in the expression of *Cd36*, *Srebf1* and *Hnf4a* genes among the four groups (data not shown).

## 4. Discussion

It is well known that consumption of diets rich in energy but poor in variety and quantity of micronutrients can lead to the development of chronic non-communicable diseases (e.g., obesity, diabetes and cardiovascular disease). In a recent study in British adults, micronutrient intake from food alone was lower than recommended levels [31]. Furthermore, there is now strong evidence in the literature for a clear association between micronutrient deficiencies and obesity across all ages and geographical areas [32,33]. A recent study showed significant negative associations between BMI and serum micronutrient levels, including folate, in overweight and obese Australian adults [34], further supporting that supplementing essential micronutrients in diets may be advantageous in the context of current unhealthy dietary patterns and obesity-associated oxidative stress. In this study, we observed several benefits of the micronutrient formulation on growth, adiposity, glucose metabolism, insulin sensitivity, hepatic antioxidant capacity and hepatic lipid metabolism. Despite strong effects on weight gain in response to HFD, there was no visible toxicity associated with supplementation.

### 4.1. Effect of Supplement on Body Weight, Adiposity and Respiratory Quotient

Despite similar energy intake among ad libitum fed H and HS rats, body weight and adiposity in HS rats was maintained to a control (C and CS animals) level throughout the study. Micronutrient supplement had no significant effects on animal’s RQ, and the basal metabolic rate was lower in HS group than H group, suggesting that supplement effect on body weight was not due to change in animal basal metabolic rate. To our knowledge, no reports of reduced weight gain in humans or animals when supplemented with folate, vitamin B6, betaine, and/or zinc or their different combination exist. Two studies reported that choline supplementation reduces weight gain in rats (by up to 31%) at a dose of 37.5 g/kg diet [15] and lowers BMI in humans by up to 12% [35] without any adverse effects. This finding was also not related to reduction in food intake, and the precise mechanism of such choline action is unknown. There is very little evidence of micronutrient supplement affecting fat mass. To date, only one study has shown effect of betaine supplement on fat mass (percent fat mass lowered by 3.3%) in humans [36], and folate supplement (epididymal fat mass lowered by 46%) in mice [11]. We observed consistent lower body weights (by around 25–30%) and fat mass (by 65%) in HS group as compared to H group. Our diet formulation was based on Wolff 3SZM diet used in mice with increased vitamin B6 and lower vitamin B12 and methionine levels, and similar effects on weight were not seen [22]. Other methyl donor supplemented diets containing additional folate, B12, betaine and choline [17,18] or folate, B12, betaine, choline, methionine, and ZnSO_4_ [19] have not been associated with effects on body weight and adiposity.

### 4.2. Effect of Supplement on Glucose Metabolism and Insulin Sensitivity

The animals fed HFD developed impaired fasting glucose (higher by 13.5%) and impaired glucose tolerance. Furthermore, the increased insulin release during OGTT, and the higher HOMA-IR values in HFD group compared to control suggest that the observed glucose intolerance may be associated with insulin resistance in HFD group [37]. Interestingly, HS animals demonstrated lower fasting glucose and insulin compared to H group. Moreover, based on insulin release (88% larger AUC for insulin release in H group as compared to HS group) and calculated HOMA-IR values (125% higher in H group as compared to HS group) as surrogate measures of insulin resistance, it is apparent that insulin resistance developed by HFD feeding may be reduced by addition of a micronutrient supplement to the HFD.

Previously, folate supplementation was reported to improve glucose metabolism in mice at a dose of 26 mg/kg diet [10] and reduce insulin resistance in rats at 40 mg/kg diet [38]. These levels are significantly higher than the 15 mg/kg used in our current study (including the 2.5 mg/kg contained in standard AIN93). In several mice studies, betaine supplementation at a dose of 1% weight/volume in drinking water improved glucose intolerance and insulin resistance [14,39,40]. Zinc supplement at twice the dose in control diet was found to improve fasting glucose and insulin sensitivity, as well as increase insulin level in diabetic rats [41]. Our supplement contained 15 g/kg of betaine, when accounting for the intake of food for the rat and water for the mouse this level of supplementation equates to an equivalent dose per gram of body. In contrast, in the current study, zinc was supplemented to almost three times more than the study by Barman et al., 2016. With this in mind, the positive effects seen on glucose metabolism and insulin sensitivity may be the result of the several single micronutrients providing an added benefit. However, it may also have arisen from several constituents interacting at the metabolic level to provide an unknown effect that not only increases the synthesis of the antioxidant molecule, glutathione, but also provides a direct improvement in glucose homeostasis. To the best of our knowledge there is no report of vitamin B6 supplement affecting glucose homeostasis and insulin sensitivity in rodents, and the effects of choline supplementation on such parameters are still unclear.

### 4.3. Effect of Supplement on Hepatic Antioxidant Capacity

High concentrations of one of the supplement key ingredients, folate, were achieved in the liver of supplemented animals. Such a high level of supplement is likely to influence hepatic metabolism and antioxidant capacity. Interestingly, supplementation increased total glutathione content (oxidized form: GSSG plus reduced form: GSH) by approximately 19% in our study. Glutathione is a major antioxidant molecule and under physiological conditions, more than 98% of glutathione exists in reduced form, which is the active form [42,43]. A rat study reported a decrease in the ratio of reduced to oxidized glutathione, upon HFD feeding, and folate supplementation (252.6 mg/kg) increased the ratio of reduced to oxidized glutathione [12]. Other rodent studies have shown improvements in hepatic glutathione concentration after betaine (dose 1% weight/volume) [44] and zinc (at a dose of 0.19 and 0.38 g/kg diet) [45] supplementation. Though there is evidence that some of the supplement constituents alone can enhance glutathione production, we tested if the micronutrient supplement as a whole can boost glutathione synthesis. Potentially, our supplement seems to drive the one-carbon intermediate, homocysteine towards transsulphuration pathway leading to glutathione formation. Vitamin B6 has been shown to enhance glutathione synthesis through such mechanisms [7]. Here, we provide evidence for the first time that this specific micronutrient supplement can have a positive effect on hepatic glutathione synthesis.

Our study showed nonsignificant increases in TBARS (a common marker of oxidative stress) levels in response to HFD. This is in contrast with previous studies in rats reporting an increase in plasma oxidative stress [46] and hepatic oxidative stress [47,48] after HFD feeding. Furthermore, as our HFD was not a potent inducer of oxidative stress we were unable to determine if the supplement is able to rectify adverse TBARS levels. The levels seen are comparable to the control animals and this may be due the requirement of an optimum level of free radicals in every cell for appropriate physiological functions [49].

It is well established that oxidative stress promotes insulin resistance and alters glucose metabolism by interfering with cellular insulin signaling, activating cellular-stress response pathway and increasing pro-inflammatory response [2,3]. The liver has important contribution in maintaining whole body glutathione homeostasis, and therefore, stimulation of hepatic glutathione synthesis may improve glucose metabolism and prevent insulin resistance in HS animals.

### 4.4. Effect of Supplement on Hepatic Lipid Metabolism

HFD fed rats had high hepatic triglyceride content (~4.5% of liver). In rodents, prolonged HFD feeding can induce hepatic changes similar to the features seen in human non-alcoholic fatty liver disease (NAFLD; characterized by liver fat >5–10% of liver weight) [50]. Interestingly, supplement prevented deposition of hepatic lipid despite the consumption of HFD but without affecting the circulating triglyceride level. This provides evidence that consumption of the supplement ameliorated one of the key phenomena, hepatic lipid deposition, occurring in NAFLD. Two components of the supplement, choline and betaine have lipotropic action, preventing fat accumulation. There are several reports of prevention of hepatic lipid accumulation by micronutrient supplementation, particularly with micronutrient combinations consisting of methyl donors. In two separate studies in rats, Cordero et al. found that methyl donor supplement (containing folate, B12, betaine, and choline) in high fat sucrose (HFS) diet can prevent the HFS diet-induced lipid accumulation in liver [17,18]. In mice with NAFLD induced by a HFD over eight weeks, four weeks of dietary methyl-donor supplementation (folate, B12, betaine, choline, methionine, and ZnSO4) was able to slow the progression of hepatic steatosis [19]. Taken together, our findings are in broad agreement with the previous studies which tested the effect of multiple components of the supplement ingredients.

To examine possible mechanisms of anti-lipid accumulation effects of the supplement, we measured the expression of major hepatic genes involved in lipid metabolism. Expression level of fatty acid translocase (*Cd36*) gene, which supports the uptake of fatty acid from plasma, suggested that plasma fatty acid uptake was similar across all groups through this receptor mediated process. Interestingly, *Cpt1a* gene was upregulated by the supplemented diet, the consequence of which can be increased fatty acid oxidation, and this may have prevented triglyceride accumulation in these animals. We saw the downregulation of *Cpt1a* gene in HFD fed animals, suggesting decreased fatty acid oxidation. Such an effect of HFD has been observed in previous studies [51,52]. Only one study in the rat showed that betaine supplementation (1 mL intragastrically of 400 mg/kg concentration) reverses the inhibition of *Cpt1a* gene expression induced by HFD [53]. While there is limited previous information, our supplement may promote fatty acid catabolism by upregulating *Cpt1a* gene.

Supplementation increased the expression of *Fabp1* gene, whereas HFD decreased its expression. *Fabp1* acts as a fatty acid binder inside hepatocytes and supports fatty acid metabolism [54]. Moreover, because it can lower free fatty acid concentration and scavenge free radicals, it acts as a useful endogenous antioxidant molecule in hepatocytes [55]. Higher expression of *Fabp1* gene in supplemented than non-supplemented animals indirectly suggests a rapid turnover of lipid molecules and provides further evidence regarding the improved antioxidant status of liver.

Surprisingly, the genes involved in fatty acid synthesis, *Acaca* and *Fasn* [56], were highly expressed in supplemented animals. A similar pattern of upregulation (significantly for *Acaca* and a trend for *Fasn* gene) was seen in methyl donor supplemented rats [17]. However, in another study in mice, Dahlhoff et al. found reduced expression of *Fasn, Acc1*, and *Acc2* in methyl donor supplemented animals [19]. The varied outcomes of the different studies may be a reflection of the different supplements used and their respective concentrations. Previously in rats fed HFD, Xu et al. observed that betaine supplementation reduces hepatic lipid accumulation by enhancing hepatic lipid export as very low density lipoproteins (VLDL) and by increasing fatty acid oxidation [53]. Similar to their findings, it is possible that our supplement also affects other activities that promote lipid efflux from liver. Furthermore, we saw upregulation of *Fasn* gene in HFD-fed animals. This outcome has been found in NAFLD patients, despite excess lipid deposits, de novo fatty acid synthesis is elevated [57], and *Acaca* and *Fasn* genes are upregulated [51,58].

Our findings suggest that HFD initiates NAFLD-like changes in rat liver, and this was ameliorated by the supplement in the diet. Hepatic lipid accumulation in NAFLD is proposed to result from increased fatty acid uptake from plasma, increased de novo lipogenesis, decreased fatty acid oxidation as a result of chronic mitochondrial overload of fatty acids and oxidative stress, and diminished export of lipids as VLDL [59]. As a result of increased glutathione synthesis, improved hepatic anti-oxidative capacity may have prevented mitochondrial beta-oxidation dysfunction in supplemented animals. It is also plausible that the supplement’s antioxidant and methyl donor activities may have directly exerted the observed changes in gene expression by altering DNA methylation or oxidative DNA damage in gene promoters [8,9].

## 5. Conclusions

Our study is the first to assess the metabolic impact of a unique dietary micronutrient supplement containing a combination folate, vitamin B6, choline, betaine, and zinc in male rats. Interestingly, this dietary supplement, when combined in an obesogenic diet, reduced the negative impact of the diet on glucose metabolism, and liver metabolism. Our findings suggest that consumption of micronutrient supplement while consuming HFD may be of value. Therefore, the dietary supplement has potential to reduce the development of metabolic diseases, which could have a significant impact on reducing obesity and obesity-related metabolic disorders in males. Further studies are required to test whether similar results can be obtained in females.

## Figures and Tables

**Table 1:** Chemical composition per kilogram of C, H, CS and HS diets. The supplemented diets had additional Choline, Betaine, Folic acid, Vitamin B6 and Zinc.

Ingredients	Control Diet (C) (g/kg)	HFD (H) (g/kg)	Control Supplemented (CS) (g/kg)	HFD Supplemented (HS) (g/kg)
Casein	140	140	140	140
Sucrose	100	405	100	405
Canola Oil	40	50	40	50
Lard		190		190
Cellulose	50	50	50	50
Corn starch	620	115	620	115
DL Methionine	3	3	3	3
Mineral mix (AIN93M)	35	35	35	35
Vitamin mix (AIN93)	10	10	10	10
Choline bitartrate *	4.1	4.1	36.5	36.5
Betaine *			15	15
Folic acid *			0.013	0.013
Pyridoxine HCl (Vit B6) *			0.0295	0.0295
ZnSO4.H2O *			0.296	0.296
Energy value	12,720 kJ/kg	12,720 kJ/kg	20,439 kJ/kg	20,439 kJ/kg

* additional to AIN93M vitamin mix.

**Table 2:** Genes selected for RT-qPCR in the liver.

Gene Names	Applied Biosystems Assay ID
Reference Genes	
*B2M*, Beta-2- microglobulin	Rn00560865_m1
*Hprt1*, hypoxanthine phosphoribosyl transferase	Rn01527840_m1
*Rplpo*, Ribosomal Protein Lateral Stalk Subunit P0	Rn03302271_gH
*Ywhaz*, tyrosine 3-monooxygenase/tryptophan 5- monooxygenase activation protein, zeta polypeptide	Rn00755072_m1
Genes of interest	
*Cd36*, Fatty acid translocase	Rn00580728_m1
*Fabp1*, Fatty acid binding protein 1	Rn00664587_m1
*Acaca*, Acetyl-CoA carboxylase alpha	Rn00573474_m1
*Fasn*, Fatty acid synthase	Rn01463550_m1
*Cpt1a*, Carnitine palmitoyl transferase 1A	Rn00580702_m1
*Hnf4a*, Hepatocyte nuclear factor 4 alpha	Rn04339144_m1
*Srebf1*, Sterol regulatory element binding transcription factor 1	Rn01495769_m1

**Table 3:** Plasma biochemical parameters.

	C	CS	H	HS
At 22 weeks				
Blood glucose (mmol/L)	5.2 ± 0.1	5.3 ± 0.1	5.9 ± 0.1 ^a^	5.5 ± 0.2 ^d^
Insulin (ng/mL)	0.68 ± 0.07	0.56 ± 0.06	1.77 ± 0.20 ^a^	0.82 ± 0.10 ^d^
HOMA-IR	3.9 ± 0.5	3.4 ± 0.4	11.7 ± 1.4 ^a^	5.2 ± 0.8 ^d^
OGTT AUC: Glucose (mM·min)	996 ± 15	1042 ± 38	1137 ± 35 ^a^	1085 ± 28
OGTT AUC: Insulin (ng/mL·min)	262 ± 22	209 ± 11	623 ± 64 ^a^	331 ± 33 ^b,d^
At 30.5 weeks				
Triglyceride (mg/mL)	0.51 ± 0.04	0.45 ± 0.04	0.72 ± 0.05 ^a^	0.67 ± 0.07 ^b^
Cholesterol (mg/mL)	0.70 ± 0.04	0.96 ± 0.05 ^c^	0.84 ± 0.06	0.86 ± 0.04
Leptin (ng/mL)	5.7 ± 0.4	5.1 ± 0.6	16.4 ± 1.8 ^a^	7.6 ± 1.0 ^d^
TBARS (µmol/L)	8.3 ± 0.4	8.6 ± 0.6	9.7 ± 0.7	9.4 ± 0.6

Data are shown as mean ± SEM (*n* = 15–17 per group). Abbreviations: C, Control diet; CS, Control diet with supplement; H, HFD; HS, HFD with supplement; OGTT, Oral glucose tolerance test; and AUC, Area under curve. Effects of HFD and supplement were assessed by two-way ANOVA followed by LSD post hoc. There was an interaction effect of HFD and supplement for blood glucose (*p* < 0.05), insulin (*p* < 0.01), HOMA-IR (*p* < 0.001), AUC: insulin (*p* < 0.01), cholesterol (*p* < 0.05) and leptin (*p* < 0.001). Overall HFD effect *p* < 0.01 or *p* < 0.001 for insulin, HOMA-IR, AUC: glucose and insulin, plasma triglyceride and leptin Overall supplement effect *p* < 0.001 for plasma insulin, OGTT AUC: insulin and leptin Post hoc HFD effect ‘a’ (H versus C) and ‘b’ (HS versus CS), and supplement effect ‘c’ (CS versus C) and ‘d’ (HS versus H).

**Table 4:** Hepatic parameters at endpoint (30.5 weeks).

	C	CS	H	HS
Folate (ng/mg)	29.3 ± 1.7	38.3 ± 2.3 ^c^	29.3 ± 1.2	35.4 ± 2.4 ^d^
Glutathione (nmol/mg)	4.8 ± 0.3	5.7 ± 0.4	4.6 ± 0.3	5.5 ± 0.3
TBARS (nmol/g)	92.3 ± 12.2	93.6 ± 9.0	109.8 ± 10.7	97.4 ± 13.0
Triglycerides (µg/mg)	31.1 ± 2.3	20.7 ± 1.4 ^c^	44.7 ± 6.3 ^a^	23.7 ± 1.3 ^d^
Cholesterol (µg/mg)	5.2 ± 0.3	4.5 ± 0.3	5.4 ± 0.4	6.1 ± 0.6 ^b^

Data are shown as mean ± SEM (*n* = 16–17 per group). Abbreviations: C, Control diet; CS, Control diet with supplement; H, HFD; and HS, HFD with supplement. Effects of HFD and supplement were assessed by two-way ANOVA followed by LSD post hoc. Overall HFD effect *p* < 0.05 for triglyceride (TG). Overall supplement effect *p* < 0.05 or *p* < 0.001 for folate, total glutathione, TG content and total cholesterol. Post hoc HFD effect ‘a’ (H versus C) and ‘b’ (HS versus CS), and supplement effect ‘c’ (CS versus C) and ‘d’ (HS versus H).

## Data Availability

Not applicable.
